# Modulation of Glycine Receptor-Mediated Pain Signaling *in vitro* and *in vivo* by Glucose

**DOI:** 10.3389/fnmol.2019.00280

**Published:** 2019-11-22

**Authors:** Rama Ashraf Hussein, Marwa Ahmed, Hans-Georg Breitinger, Ulrike Breitinger

**Affiliations:** Department of Biochemistry, German University in Cairo, New Cairo, Egypt

**Keywords:** nociception, spinal α3 glycine receptors, glycinergic transmission, glucose modulation, patch-clamp recording, pain-related behavior, tactile sensitivity, hotplate assay

## Abstract

The inhibitory glycine receptor (GlyR) plays an important role in rapid synaptic inhibition in mammalian spinal cord, brainstem, higher brain centers, and is involved in transmission of nociceptive signals. Glucose and related mono- and disaccharides potentiate currents mediated by recombinant α1, α1-β, and α3 GlyRs. Here, we confirmed the specific potentiation of α3 GlyR signaling by glucose through: (i) patch-clamp electrophysiology on recombinant receptors; and (ii) by verifying *in vitro* data in a mouse model *in vivo*. Mice were intraperitoneally (IP) injected with glucose (2 g/kg) or vehicle, and then challenged with sublethal doses of strychnine (0.2 mg/kg and 0.5 mg/kg). Pain-related behavior was assessed using two established models: (i) touch sensitivity tests using von Frey filaments; and (ii) hotplate assay. We observed a reduction of pain sensitivity in glucose-treated mice relative to vehicle-treated control mice. Injection of strychnine resulted in an increased sensitivity to tactile and heat stimuli, which was reversed in the presence of glucose. Analgesic effects of glucose were more pronounced in von Frey experiments, consistent with the established use of this model for neuropathic pain. Overall, glucose showed mild analgesic effects and was able to compensate for strychnine-induced allodynia in mice. Since the action of strychnine is specific for GlyR, these experiments show for the first time an *in vivo* potentiation of GlyR activity by glucose and suggest a molecular mechanism for glucose-mediated analgesia.

## Introduction

**Figure 1:**
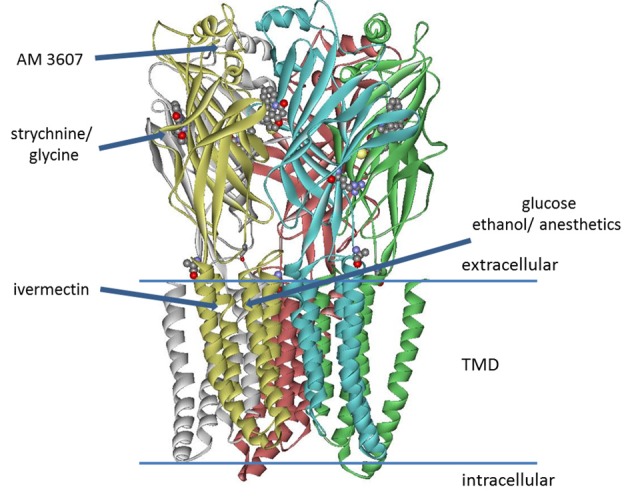
Model of glycine receptor (GlyR), adapted from pdb data of the zebrafish α1 electron cryo-microscopy structure (Du et al., 2015). The binding site of AM-3607 was identified for GlyR α3 (Huang et al., 2017). Strychnine and the side chain of residue Lys143 (not fully resolved in the structure) are represented as ball and stick structures. Binding of strychnine, glycine, ivermectin and AM-3607 was determined from high-resolution structures, while the sites for ethanol, anesthetics (Mihic et al., 1997; Lobo et al., 2004), as well as the putative glucose binding site (Breitinger et al., 2016) were postulated from mutagenesis studies and functional analysis.

The inhibitory glycine receptor (GlyR) is one of the principal mediators of rapid synaptic inhibition in the mammalian spinal cord, brainstem, and higher brain centers. It is a member of the Cys-loop family of ligand-gated ion channel receptors, which includes nicotinic acetylcholine; GABA_A_ and serotonin type 3 (5-HT_3_) receptors. Cys-loop receptors are pentameric transmembrane complexes that surround a central ion pore which opens transiently upon binding of the activating ligand. In the case of the GlyR, five subunits (α1–4, β) have been identified. GLRA4, encoding the α4 subunit is a pseudogene in humans, while α2 receptors are predominant in neonatal tissue and their abundance decreases dramatically in most adult tissues (Breitinger, 2014). α1-β receptors are mainly located in brainstem and spinal cord, where they mediate muscle tone and movement; they have also been found in numerous other tissues including the mesolimbic system (Munoz et al., 2018), while α3 subunits are found in higher brain centers and spinal cord where their prominent contribution to nociceptive signal transmission has been well established (Harvey et al., 2004, 2009; Lynch and Callister, 2006; Zeilhofer et al., 2012). In the last years, several high-resolution structures became available that provide important information about binding sites on the receptor, including electron microscopy studies on Zebrafish α1 GlyR bound to strychnine, glycine, and ivermectin (Du et al., 2015), the crystal structure of GlyR α3 exhibiting the binding site of strychnine (Huang et al., 2015), and recently the structure of α3 receptor bound to a novel class of analgesic GlyR potentiators (Huang et al., 2017; Figure 1). This study identified a novel binding site for analgesics near the N-terminus of the receptor protein. In addition to well-defined sites from GlyR structures, binding sites for ethanol and anesthetics—both enhancing GlyR function—had been postulated earlier. Using chimeric receptor constructs and subsequent mutagenesis studies identified Ser267 as a crucial amino acid residue for their action (Mihic et al., 1997). Specific amino acids enhancing the effects of alcohol and anesthetics were suggested to border a water-filled cavity between transmembrane domains 2–3 (Mihic et al., 1997; Lobo et al., 2004). This binding pocket was thought to be present in all cys-loop receptors, yet the modulatory effects of alcohols and anesthetics vary between different members of this receptor family (Dupre et al., 2007). Glucose, a polyalcohol, may indeed be able to access this cavity on the receptor.

Loss-of-function mutations in GlyR genes underlie complex motor disorders, such as human hyperekplexia (STHE, startle disease, OMIM 149400), stiff-man syndrome, bovine myoclonus, and other neuromuscular diseases. GlyR function is inhibited specifically by strychnine but also by other compounds including brucine (Pullan and Powel, 1992), ginkgolide B (Kondratskaya et al., 2004) or flavonoids (Huang and Dillon, 2000; Zhu et al., 2003; Raafat et al., 2010). Biphasic modulation is observed for Zn^2+^ (Bloomenthal et al., 1994). Positive modulation has been described for glutamate (Liu et al., 2010), ethanol and anesthetics (Mascia et al., 1996). Glucose and related mono- and disaccharides were recently identified as positive modulators of recombinant α1, α1-β (Breitinger et al., 2015), and α3 (Breitinger and Breitinger, 2016) GlyRs in HEK293 cells, reducing EC_50_ value 2–4 fold. When glucose was present in the cell culture medium for 2–24 h prior to the experiments, positive modulation was observed even in the absence of sugar during experiments (Breitinger et al., 2015, 2016). Other sugars, including fructose, mannose, and the disaccharides lactose and sucrose also potentiate GlyR currents (Breitinger et al., 2016). EC_50_ of glucose and fructose for augmentation of glycine-mediated responses was ~6–7 mM. At sugar concentrations >10 mM, the maximum of current enhancement was reached within ~30 min, corresponding on-rates were <0.5 h for saturating concentrations of monosaccharides (glucose and fructose) and ~1.5 h for disaccharides (sucrose and lactose). Off-rates were considerably slower (>24 h). Kinetics and concentration dependence of receptor potentiation are similar to those of protein glycation, suggesting a possible mechanism for GlyR modulation by sugars (Breitinger et al., 2016).

After overnight preincubation with 10 mM of glucose, we observed the maximum potentiation of recombinant α1 (Breitinger et al., 2016) and α3 (Breitinger and Breitinger, 2016) GlyRs. Fasting glucose levels in healthy individuals are ≤ 5.6 mM (≤100 mg/dl), levels of 5.6–7.9 mM (100–125 mg/dl) are considered prediabetic, and 7 mM (126 mg/dl) or higher indicate diabetes^1^. Thus, GlyR modulation is observed at physiological levels of glucose.

Since α3 GlyRs are involved in pain signaling pathways, we investigated the influence of glucose on *in vivo* pain-related behavior in two different mouse models, namely von Frey filaments and hotplate tests. Both experiments demonstrated a significant reduction of touch and pain sensitivity of mice after glucose treatment.

## Materials and Methods

### Cell Culture and Transfection

HEK293 cells were grown, passaged and transfected using PEI as described (see Supplementary Materials).

### Electrophysiological Recordings and Data Analysis

Current responses from GlyR-transfected HEK293 cells were measured at room temperature (21–23°C) at a holding potential of −50 mV using a HEKA EPC10 amplifier (HEKA Electronics, Lambrecht, Germany) controlled by Pulse software (HEKA Electronics). Recording pipettes were pulled from borosilicate glass (World Precision Instruments, Berlin, Germany) using a Sutter P-97 horizontal puller (Sutter, Novato, CA, USA). Solutions were applied using an Octaflow system (NPI electronics, Tamm, Germany). The external buffer consisted of 135 mM NaCl, 5.5 mM KCl, 2 mM CaCl_2_, 1.0 mM MgCl_2_, and 10 mM Hepes (pH adjusted to 7.4 with NaOH); the internal buffer was 140 mM CsCl, 1.0 mM CaCl_2_, 2.0 mM MgCl_2_, 5.0 mM EGTA, and 10 mM Hepes (pH adjusted to 7.2 with CsOH). Glucose (Sigma-Aldrich, Munich, Germany) was added to the growth medium on the day before experiments to give a pre-exposure to 50 mM of glucose for 16–20 h. Dose-response data were fitted to the Hill equation (see Supplementary Material) to determine EC_50_ and IC_50_. Significance of differences between EC_50_ values were determined using one-way ANOVA with *p* ≤ 0.05 (*) and *p* ≤ 0.01 (**) taken as significant.

### Animal Housing

All animal experiments were performed according to ARRIVE guidelines and approved by the Ethics committee of the German University in Cairo. Animals were not sacrificed during or after any experiment. Male Swiss Webster mice weighing 20–45 g were used in this experiment. Four to eight animals were housed together in standard mouse cages with free access to chow and water with a 12 h light/dark cycle. Littermates were divided equally between experimental groups.

### Tactile and Heat Sensitivity Tests

See Supplementary Material S1 for experimental details. On the day of the experiment, mice were weighed and then fasted for 5–6 h to reduce variability in initial blood glucose levels (BGL). Mice had been accustomed to the test apparatus for 2–3 h on two separate days before experiments. Two hours before testing, mice were acclimatized to the measuring apparatus. For hotplate tests, mice were accustomed to the testing condition by placing them one by one on a switched off hotplate at room temperature for the set cut off time of 30 s. One hour before testing, baseline measurements for touch sensitivity were recorded. For both experiments, mice were then injected according to protocol. Blood glucose level was measured before and after injections (Figure 3A).

**Figure 2:**
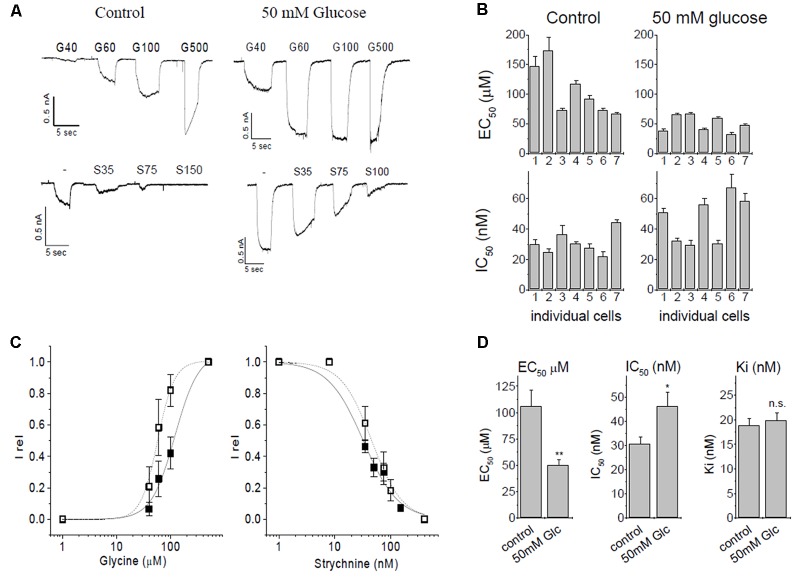
Patch-clamp studies of α3 GlyR modulation by glucose and strychnine. **(A)** Glycine induced currents in HEK293 cells transfected with GlyR α3L constructs. Top panels: currents from untreated controls (5.5 mM glucose) and after overnight preincubation in 50 mM glucose. Bottom panels: inhibition by strychnine in the presence of 60 μM glycine of untreated cells, or cells preincubated with 50 mM glucose. **(B)** EC_50_- and IC_50_ values of seven individual cells without (control) and with glucose preincubation. **(C)** EC_50_ and IC_50_ curves for control conditions (solid squares, solid line) and after preincubation with 50 mM glucose (open squares, dashed line). **(D)** Summary of changes of ion channel parameters before (left bar) and after (right bar) pretreatment with 50 mM glucose. Left panel: EC_50_ values for glycine. Middle panel: IC_50_, values of strychnine at 60 μM glycine. Right panel: K_i_ for strychnine at 60 μM glycine. K_i_ was calculated using the Cheng-Prusoff correction, significance was tested using one-way ANOVA with *p* < 0.05 considered significant. Note that the observed IC_50_ for strychnine increases after receptors were preincubated with 50 mM glucose because glucose left-shifts concentration-response curve of glycine. The Cheng-Prusoff correction considers this change in EC_50_, and K_i_ for strychnine is independent of the glucose concentration. *n* = 7 cells were used in all experiments. All data are means ± SEM; significance levels (relative to control) are indicated: *p ≤ 0.5; **p ≤ 0.01; ns, not significant.

**Figure 3:**
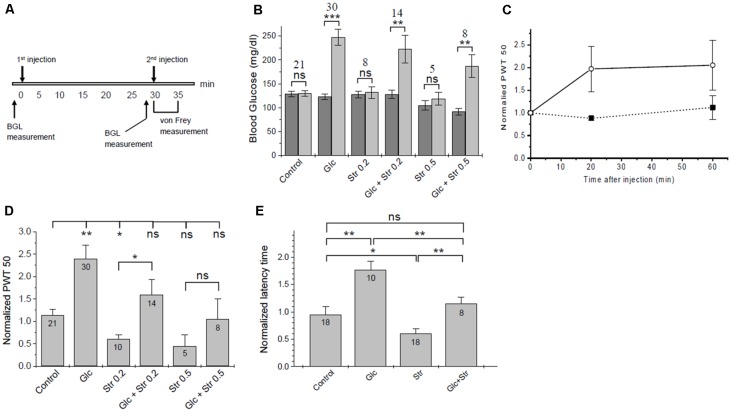
Touch- and heat sensitivity tests. **(A)** Time course of *in vivo* tests. **(B)** Changes in blood glucose. Blood glucose levels (BGL) at time 0 (dark bars) and 30 min after 1st injection (light bars) are plotted for the experimental groups. The number of animals in each group and the significance of differences in BGL before/after injection are given. Significance was tested using one-way ANOVA with *p < 0.05, **p < 0.01, ***p < 0.005, ns, not significant. See Supplementary Material S2 for a list of blood glucose values. **(C)** Time course of tactile sensitivity after glucose administration. Paw withdrawal threshold (PWT), normalized to baseline, was measured 20 min and 60 min after glucose injection (open circles, dashed line, *n* = 4) or PBS control (solid squares, solid line, *n* = 3). Analgesic effects of glucose persisted for >60 min. **(D)** Summary of normalized PWT values after treatment of mice with glucose/vehicle and different doses of strychnine. Data are given as means ± SEM, see Supplementary Material S2 for details. Significance of differences (one-way ANOVA) is indicated with *p < 0.05, **p < 0.01, ns, not significant. The number of animals for each condition is given. **(E)** Hotplate test—normalized latency time until heat-related behavior (means ± SEM). Significance is indicated (one-way ANOVA); *p < 0.05, **p < 0.01, ns, not significant. The number of animals in each experimental group is indicated.

Mice were divided into four experimental groups (see main text). The investigator performing the measurements was blinded to treatment groups and to the blood glucose readings. All mice were measured for their Paw withdrawal threshold (PWT) using von Frey filaments on at least one occasion before the testing day to reduce the stress or surprise from the experiment. We used the up-and-down method to determine pain withdrawal thresholds (PWT; Bradman et al., 2015) with 8 mN as the starting value (see Supplementary Material for details). Baseline PWT values for each mouse were determined before treatment. Indices of tactile sensitivity were calculated by dividing 50% PWT values of each mouse by the baseline values that had been before treatment. Measurements were limited to the time window of 5 min after the second injection (strychnine or vehicle) due to the fast elimination kinetics of low doses of strychnine.

For hotplate tests, mice were placed in a 500 ml glass beaker set on the hotplate at 55°C. Mice were placed one by one on the hotplate, the time for the first nocifensive response was recorded using a stopwatch then the animal was immediately removed from the hotplate. The cut off time for the test was 30 s. Recorded responses were either licking or shaking of the hind paw, vocalization, or attempting to escape. Time until the first response was normalized to a control time taken 1 h before the injection protocol was started; ratios of response times were reported and given as average ± SEM.

See Supplementary Material for full details of experiments.

## Results

### Glucose-Potentiated α3 Glycine Receptors Are Strychnine Sensitive

Initially, we verified the augmentation of recombinant α3L GlyRs expressed in HEK293 cells by glucose (Glc) and investigated the inhibition of the receptor by strychnine. In this set of experiments, the EC_50_ of control currents (no glucose) was 106 ± 16 μM, in comparison to 49.9 ± 5.3 μM after preincubation with 50 mM glucose (Figures 2A–C). The shift of EC_50_ was significant (*p* = 0.0046, one-Way ANOVA).

Strychnine is a highly specific antagonist with nanomolar affinity for the receptor. For inhibition experiments, glycine concentration was kept constant at 60 μM, which represents ~EC_25_ in control cells, and ~EC_50_ in glucose-treated cells. We tested *n* = 7 cells for each condition (Figure 2B). Strychnine inhibition at 60 μM of glycine gave IC_50_ values of 30.7 ± 2.8 nM and 46.2 ± 5.8 nM in the absence or presence of 50 mM glucose, respectively. This shift was statistically significant (*p* = 0.034). K_i_ values were calculated using the Cheng-Prusoff equation (Cer et al., 2009) to account for the different effective EC_50_ of glycine in the absence and presence of glucose, giving K_i_ of 18.8 ± 1.4 nM for control cells and 19.8 ± 1.6 nM for cells pretreated with 50 mM glucose. After correcting for the glucose-induced left-shift of the EC_50_ curve, the difference in the Ki of strychnine was below statistical significance (*p* = 0.66, one-way ANOVA). We thus concluded that inhibition of α3 GlyRs by strychnine is independent of the absence or presence of glucose (Figure 2D).

### Glucose Reduces Tactile and Heat Sensitivity in Mice

*In vivo* tests were carried out in accordance with GUC Ethics and Animal care regulations. The LD_50_ of strychnine on mice is reported to be 0.98 mg/kg for intraperitoneal (IP) injection (Prasad et al., 1981; Patocka, 2009) and 2 mg/kg for oral administration (Setnikar et al., 1960; Patocka, 2009), while 2 mg/kg of IP administered strychnine was found to be lethal in 85% of Swiss Webster mice (Maher et al., 2014). In our study, non-lethal doses of strychnine of 0.2 mg/kg and 0.5 mg/kg were used, while the glucose dose was 2 g/kg in all experiments.

### Tactile Sensitivity (von Frey) Tests

Tactile sensitivity was assessed using calibrated von Frey filaments (Department of Physiology, University of Erlangen, Germany). The protocol used was as follows (Figure 3A):

(i) Baseline PWT determination for each mouse, 30 min prior to the experiment;
(ii) at time *t* = 0, mice were injected with glucose (2 g/kg) or vehicle
(iii) at *t* = 30 min, mice were injected with strychnine (0.2 or 0.5 mg/kg) or vehicle;
(iv) at *t* = 35 min pain-related behavior was assessed.

Whenever glucose was injected, we observed a robust increase in BGL (Figure 3B, Supplementary Tables S1–S3). The timing of the strychnine injection was critical since our initial experiments showed that sub-lethal amounts of strychnine, as used here, are metabolized quickly, and after >5 min notable effects on pain sensitivity were gone. Effects of glucose on pain-related behavior were fully developed after 20 min and stable until at least 60 min post-injection (Figure 3C), in agreement with the time course for changes in BGL after IP injection (Xu et al., 2016). Thirty minutes after injection, BGL level was found to be increased by 2.2 ± 0.6 and 2.0 ± 0.8-fold in glucose and (Glc+Str) groups, respectively. There was no correlation between the magnitude of increase in BGL and the extent of glucose effect on pain hypersensitivity in Glc group mice (see Supplementary Tables S1–S3).

In agreement with previous studies (Yaksh, 1989; Lim and Lee, 2010) strychnine injected mice showed a significant increase in tactile sensitivity relative to control (Figure 3D). Upon glucose injection, PWT increased ~2-fold relative to control (Supplementary Tables S1–S3). Increasing concentrations of strychnine led to increasing touch sensitivity in both, glucose-treated mice and vehicle-treated control animals (Figure 3D). Glucose treatment compensated hypersensitivity and allodynic effects of 0.2 and 0.5 mg/kg of strychnine (Supplementary Tables S1–S3). This indicates that increased blood glucose could indeed lower pain sensitivity in mice. Furthermore, glucose counteracted strychnine-induced touch sensitivity, indicating that at least part of the analgesic effect of glucose was mediated through the glycinergic pathway.

### Hotplate Test

Hotplate tests were performed as a second assessment of pain-related behavior. Here, we measured the latency time of mice until pain-related reaction on a 55°C hotplate. Groups and injection protocols were as described for tactile sensitivity tests. Following the second injection, the latency time on the hotplate was recorded and normalized to baseline time. Glucose exhibited an analgesic effect with a significant increase of the average normalized latency time from 0.99 ± 0.10 (control) to 1.79 ± 0.18 (+glucose). Strychnine decreased the thermal pain threshold, causing a statistically significant decrease in the average normalized latency time of mice on the hotplate compared to control (Figure 3E). Normalized time to response was 0.63 ± 0.10 for mice injected with 0.2 mg/kg strychnine. Mice who received glucose pretreatment followed by 0.2 mg/kg of strychnine had a normalized response time of 1.72 ± 0.44. Mice who received 0.5 mg/kg strychnine showed a further reduction of normalized response time (0.30 ± 0.07), which was increased to 0.74 ± 0.15 in mice who received 2 g/kg of glucose prior to the strychnine challenge. Thus, increased heat sensitivity caused by strychnine was offset after pretreatment of mice with glucose in a dose-dependent manner, in agreement with the results from von Frey tactile tests (Figure 3D). Changes in heat sensitivity by glucose (reduced) and strychnine (increased) were statistically significant, while heat-related behavior of mice receiving both compounds was not different from control animals (Figure 3E).

## Discussion

Glycinergic interneurons control the transmission of these afferent nerve fibers to the projection neurons that ascends to the brain thereby controlling pain transmission (Zeilhofer and Zeilhofer, 2008; Imlach et al., 2016). Alpha3 GlyRs inhibit Aβ fibers from amplifying nociceptive signal pathways (Lynch and Callister, 2006; Harvey et al., 2009; Zeilhofer et al., 2012). Inhibition of α3 GlyR activity removes this block, the resulting “disinhibition” allows non-noxious signals from Aβ fibers to enter pain circuitry, and these signals are then transmitted to the brain as noxious, causing allodynia and hypersensitivity (Zeilhofer et al., 2012). This type of allodynia is observed in some forms of neuropathy or caused by specific GlyR blockers such as strychnine (Imlach et al., 2016), or prostaglandins which reduce α3 GlyR activity through EP2 receptors and protein kinase A (Harvey et al., 2004). The increase in sensitivity to tactile stimuli after administration of strychnine is well documented in the literature. Our results agree well with reports showing that strychnine injection increased pain hypersensitivity in von Frey test due to its blockade of synaptic GlyR ion channels (Lim and Lee, 2010). Moreover, it is well established that spinal glycinergic interneurons are important in controlling and processing tactile sensation. In many studies investigating neuropathic pain, loss of glycinergic inhibition on Aβ afferent fibers was implicated in perceiving tactile (non-painful) sensation as pain (Sivilotti and Woolf, 1994; Lim and Lee, 2010; Imlach et al., 2016).

The opposite effect, analgesia, is observed upon GlyR stimulation by cannabinoids (Ahrens et al., 2009; Xiong et al., 2011, 2012), showing that α3 receptor activity is an essential component of both, augmentation and suppression of nociceptive signals. The ability of glucose to offset strychnine-induced hypersensitivity is consistent with a direct augmentation of α3 GlyR function by glucose, as was observed *in vitro* by patch-clamp studies (Breitinger and Breitinger, 2016). Notably, glucose was shown to potentiate not only α3 but also α1 and α1-β GlyRs (Breitinger et al., 2015, 2016). Indeed, similar effects were reported for cannabinoids (Ahrens et al., 2009; Wells et al., 2015). Indeed, cannabinoid-related ligands for α1 receptors were used for a virtual screening approach in search of novel analgesics (Wells et al., 2015). The dependence of glucose-mediated analgesia on strychnine concentration that we observed is a strong argument in favor of a specific action of glucose on α3 GlyRs *in vivo*. The observed analgesic effects of glucose would thus be the result of: (i) a reduced efficiency of strychnine because of the left-shift of the EC_50_ of glycine at higher glucose concentrations; and (ii) a potentiation of uninhibited GlyR currents, compensating the loss of strychnine-inhibited receptors. Apparently, glucose binding generates a more active receptor with decreased apparent strychnine sensitivity when measurements are performed at the same concentration of glycine. Converting IC_50_ values into K_i_ binding constants—thereby correcting for the reduced EC_50_ values in presence of 50 mM glucose—shows that strychnine inhibition is not affected by glucose.

An interplay between glucose and neuronal ion channels, as well as crosstalk between glucose and glycine metabolism has been reported. Indirect effects of glucose concentration on leptin-induced IPSCS and GABAergic modulation in insular cortex cells were described (Murayama et al., 2019). Indeed, GABA_A_ receptors contribute to the activity of pancreatic beta cells (Korol et al., 2018), but no direct modulation of the receptor by glucose was reported. Glycine itself was reported to be an antioxidant and protective of pancreatic cells in diabetic rats (Chen et al., 2018), the effects of glycine on glucose homeostasis (but not vice versa) have recently been reviewed (Yan-Do and MacDonald, 2017). Ketone bodies have been shown to modulate ligand-gated in channel function, with β-hydroxybutyrate at physiological concentrations being an inhibitor of GlyRs expressed on Xenopus oocytes (Pflanz et al., 2019).

Structural data reveal the binding sites of strychnine, glycine, ivermectin and a new class of analgesic potentiators AM-3607 (Du et al., 2015; Huang et al., 2015, 2017). Considering these structures, the distance between residue Lys143—putatively involved in glucose binding—and the glycine- or strychnine pocket is too large for direct interaction. For the same reason, there is no direct evidence to suggest a newly discovered analgesic binding site close to the N-terminal α-helix (Huang et al., 2017) as glucose binding pocket. However, the binding cavity of ivermectin from electron cryo-microscopy data (Du et al., 2015) is close to Lys143. The key residue postulated for the action of alcohol and anesthetics is Ser267 (Mihic et al., 1997; Lobo et al., 2004), which is also involved in ivermectin binding (Du et al., 2015), suggesting that these sites overlap. Ivermectin and volatile anesthetics are hydrophobic, yet hydrophilic alcohols are also postulated to bind to the same pocket (Mihic et al., 1997; Lobo et al., 2004), which appears to accommodate both, hydrophilic and hydrophobic compounds. It is noted, that the side chain of Lys143 was not resolved in two high-resolution structures (Du et al., 2015; Huang et al., 2015). This is indicative of high conformational flexibility, which is generally considered a good prerequisite for binding (Najmanovich et al., 2000; Zavodszky and Kuhn, 2005). While the interaction of ivermectin with the receptor has been resolved, structural data of the glucose-bound receptor are not yet available. Similar interactions between M3 of the (+) subunit, M1 of the (−) subunit, and contacts with M2 and M2/M3 loop can be inferred, but require experimental proof.

Diabetic neuropathy (DN) is a common complication of diabetes, where the patients’ pain threshold can be increased or decreased. Non-painful DN represents a clinical and diagnostic challenge, often going unnoticed until irreversible nerve damage has occurred (Gylfadottir et al., 2019). Over time, at least 50% of patients with diabetes develop DN. Although overall understanding of the complexities of DN has substantially evolved over the past decade, the distinct mechanisms underlying neuropathy in type 1 and type 2 diabetes remains unknown (Feldman et al., 2019). Tight control of hyperglycemia reduces the incidence of DN in type 1 diabetes mellitus but its role in type 2 diabetes is less clear. The cause of DN remains controversial, focused on the impact of metabolic abnormalities, polyol flux, microvascular changes, mitochondria, oxidative stress, lipid biology and others (Zochodne, 2019). Glycemic control is known to reduce diabetic neuropathic pain. However, it was observed that too severe reduction of BGL increases the risk of hypoglycemic episodes, which are themselves linked to painful neuropathy (Zhang et al., 2016). This phenomenon, known as “insulin neuritis” or “treatment-induced neuropathy,” has been reported in both type 1 and type 2 diabetic patients treated with insulin or oral hypoglycemic agents, who typically had a history of poor glycemic control (Gibbons and Freeman, 2010; Knopp et al., 2013). The involvement of GlyR α3 signaling in pain regulation could explain these phenomena. In the case of non-treated diabetes, BGL are usually elevated, thereby increasing GlyR function and one would expect reduced pain sensitivity. Under hypoglycemic conditions, GlyR activity would be decreased and pain sensitivity increased.

To our knowledge, a direct effect of glucose-induced GlyR potentiation (Breitinger et al., 2015, 2016) on pain sensitivity *in vivo* has not been described before. However, a recent study has shown that morphine-mediated analgesia in rats is enhanced upon intraperitoneal administration of glucose, and is not related to glucose “taste or gustation” (Yamamoto et al., 2014), suggesting a “molecular” effect of glucose, similar to our observations. Indeed, potentiation of α3 GlyR activity by glucose could enhance other mechanisms of analgesia. Combined evidence from *in vitro* and *in vivo* studies suggests that glucose has a direct effect on nociceptive signaling *via* potentiation of spinal α3 GlyRs, and modulation of neuronal ion channels by glucose may indeed be a relevant physiological mechanism. Data suggest the α3 GlyR signaling pathway as a possible player in DN.

## Data Availability Statement

All datasets generated for this study are included in the article/Supplementary Material.

## Ethics Statement

The animal study was reviewed and approved by Ethics Committe, German University in Cairo.

## Author Contributions

UB and H-GB devised the study and wrote the manuscript. RH, MA and UB devised and performed experiments. RH, MA, UB and H-GB analyzed the data.

## Conflict of Interest

The authors declare that the research was conducted in the absence of any commercial or financial relationships that could be construed as a potential conflict of interest.

## Footnotes

^1^https://www.mayoclinic.org/diseases-conditions/diabetes/diagnosis-treatment/drc-20371451
