# Supplementary material for: Modulation of Glycine Receptor-Mediated Pain Signaling in vitro and in vivo by Glucose
Source: Front Mol Neurosci. 2019 Nov 22;12:280. doi: 10.3389/fnmol.2019.00280 (PMC6883931; doi:10.3389/fnmol.2019.00280)
Supplement: Supplementary file 1 [file Data_Sheet_1.docx]

**Modulation of glycine receptor-mediated pain signalling**

**in vitro and in vivo by glucose**

Rama Ashraf Hussein, Marwa Ahmed, Hans-Georg Breitinger* and Ulrike Breitinger*

**Supplementary Material S1**

**Materials and Methods – Details**

*Cell Culture and Transfection* – HEK293 cells were grown in 10 cm tissue culture Petri dishes in Eagle minimal essential medium (EMEM) (Lonza, Basel, Switzerland) supplemented with 10% FBS (Invitrogen, Karlsruhe, Germany) and penicillin/streptomycin (Sigma-Aldrich, Munich, Germany) at 5% CO_2_ and 37 °C in a water saturated atmosphere. For electrophysiological experiments, cells were plated on acetone treated glass coverslips in 24 well plates. Transfection was performed 1 day after cell passage using polyethyleneimine (PEI) (Sigma-Aldrich, Munich, Germany): 0.75 µg of receptor DNA and 1.0 µg of green fluorescent protein DNA were mixed in 50 µl EMEM; in a second Eppendorf tube, 3.5 µl PEI was added to 50 µl EMEM, mixed and combined with the DNA solution. After 20 min incubation at room temperature, the transfection solution was added dropwise to the cells. Measurements were performed 1-3 days after transfection.

*Electrophysiological Recordings and Data Analysis* – Current responses from GlyR-transfected HEK293 cells were measured at room temperature (21-23°C) at a holding potential of ‑50 mV. Whole-cell recordings were performed using a HEKA EPC10 amplifier (HEKA Electronics, Lambrecht, Germany) controlled by Pulse software (HEKA Electronics). Recording pipettes were pulled from borosilicate glass (World Precision Instruments, Berlin, Germany) using a Sutter P-97 horizontal puller (Sutter, Novato, CA). Solutions were applied using an Octaflow system (NPI electronics, Tamm, Germany), where cells were bathed in a laminar flow of buffer, giving a time resolution for solution exchange and re-equilibration of about 100 ms. The external buffer consisted of 135 mM NaCl, 5.5 mM KCl, 2 mM CaCl_2_, 1.0 mM MgCl_2_, and 10 mM Hepes (pH adjusted to 7.4 with NaOH); the internal buffer was 140 mM CsCl, 1.0 mM CaCl_2_, 2.0 mM MgCl_2_, 5.0 mM EGTA, and 10 mM Hepes (pH adjusted to 7.2 with CsOH). Glucose (Sigma-Aldrich, Munich, Germany) was added to the growth medium as indicated. Dose response data was fitted to the Hill equation

$$I_{glycine}= I_{sat}\times\frac{\left[ glycine \right]^{n_{H}}}{{EC}_{50}^{n_{H}}+ \left[ glycine \right]^{n_{H}}}$$

using a nonlinear algorithm in Microcal Origin. Here, I_glycine_ is the current amplitude at a given glycine concentration, I_sat_ is the maximum current amplitude at saturating concentrations of glycine, EC_50_ is the glycine concentration at half-maximal current responses, and n_H_ is the Hill coefficient. Currents from each individual cell were normalized to the maximum response at saturating glycine concentrations. IC_50_ curves were fitted using the equation

$$I_{inh}= I_{glycine}\times\frac{{IC}_{50}^{n_{H}}}{{IC}_{50}^{n_{H}}+\left[ inh \right]^{n_{H}}}$$

where I_inh_ is the current in presence of inhibitor, I_glycine_ is the current in absence of inhibitor, IC_50_ is the inhibitor concentration that causes 50 % inhibition, and n_H_ is the coefficient for inhibition. Currents from each individual cell were normalized to the maximum response at saturating glycine concentrations. Significance of differences between EC_50_ values were determined using one-way ANOVA with p ≤ 0.05 (*) and p ≤ 0.01 (**) taken as significant. Whole-cell recordings were taken in glucose-free extracellular buffer, where GlyR-transfected HEK293 cells had been exposed to increased sugar (50 mM glucose, control: 5.5 mM glucose) in the culture medium for 16 hours. In all experiments EC_50_ values were determined for each individual cell from a non-linear fit of dose response data to the logistic equation (above). An un-weighted average was calculated from all individual EC_50_ values, without considering the fitting errors. All data are given as means ± standard error of the means. For calculation of K_i_, the Cheng-Prusoff correction was used (Cer et al., 2009):

$$K_{i}=\frac{{IC}_{50}}{1+\frac{[Gly]}{{EC}_{50}}}$$

*Animal Housing* – Male Swiss Webster mice weighing 20-45 g were used in this experi­ment. The animals were obtained from the breeding colony of the national research center, Cairo, Egypt. 4-8 animals were housed together in standard mice cages and given free access to chow and tap water. They were kept in a 12 h light/dark cycle. Littermates were divided equally between experimental groups.

*Mice treatment on the day of experiment* – On the day of the experiment, mice were weighed and then fasted for 5-6 hrs in the morning to reduce variability in initial blood glucose levels. Longer fasting periods were not employed since they may change the metabolic profile of mice and also may trigger changes in anti-insulin hormones which might have unpredictable effects on both blood glucose levels (Ayala et al., 2010) and cortisol levels that might by itself directly affect pain sensation (Maiaru et al., 2016). In case of von Frey experiment, mice were acclimatized to the measuring apparatus two hours before testing by placing each mouse under a 250 ml beaker on a raised stand with mesh floor. Moreover, mice are acclimatized to the testing conditions by leaving them in the measuring apparatus for 2-3 hours on two different days before the experiment. For the hot plate test, mice were acclimatized to the testing condition by placing them one by one on a switched off hotplate at room temperature for the set cut off time of 30 sec. One hour before testing, baseline measurements were recorded. For both experiments, mice were then injected following the injection protocol. Mice were monitored for blood glucose level (BGL) directly before the 1st injection and the 2nd injection to confirm a rise in blood glucose level of mice receiving intraperitoneal glucose compared to the ones receiving PBS. Blood samples were collected for that purpose by a needle incision or pricking in the tail vein. Blood Glucose level was measured using a FAD-glucose dehydrogenase assay glucose meter (Glucodoctor super sensor, Mekkawy, Cairo).

*Von Frey Experiment –* The investigator performing the measurements was blinded to treatment groups and to the blood glucose readings. All mice were measured for their paw withdrawal threshold using the Von Frey filaments on at least one occasion before the testing day. That was done to reduce the stress or surprise that might result from exposure to new conditions on the day of the experiment. A baseline reading for each mouse is also taken before the test with the consideration to leave at least one-day gab between baseline and experiment measurement to minimize any effect of repetitive measurement. The Von Frey test takes place by applying von Frey hair No. 4 (8 mN) following the up and down method within 5 min of the second injection (saline or strychnine). The Paw withdrawal threshold (PWT) values are then normalized to the values before treatment. Pairwise One-way ANOVA was performed to detect statistically significant differences between groups. All pain-related behavioural measurements were limited to the time window of 5 min due to the fast elimination kinetics of low dose of strychnine.

We used the up-and-down method to determine pain withdrawal thresholds (PWT) (Bradman et al., 2015). To assess the starting force, baseline 50% PWT was measured in five naïve mice (20-35 g) by application of von Frey hairs in an ascending order of their applied force. It was found that the 50% PWT in naïve mice was around 8-11.4 mN. Consequently, 8 mN was used as starting value in tactile sensitivity tests. Baseline PWT values for each mouse were determined before treatment. The 50% PWT was estimated using maximum likelihood values after Dixon, modified by Chaplan et al. (Dixon, 1965; Chaplan et al., 1994). Indices of tactile sensitivity were calculated by dividing 50% PWT values of each mouse by the baseline values that had been before treatment. Results from several runs were pooled and averaged.

*Experimental groups in von Frey test* – Mice were divided into four experimental groups. The control group mice were injected twice with PBS with a time lapse of 30-35 min in between. The glucose group mice received an intraperitoneal injection of glucose in PBS (2g/kg), followed by an injection of PBS after 30-35 min. The strychnine group mice were intraperitoneal injected with PBS, while after 30-35 min injection with strychnine in PBS (0.2 mg/kg) followed. In the glucose and strychnine group, mice received an intraperitoneal injection of glucose (2g/kg), followed by an injection of strychnine (0.2 mg/kg) after 30-35 min. All experimental groups received a uniform number of injections and hence any possible effect of multiple injections on tactile sensitivity would be equal between groups.

*Hotplate Assay* – Hotplate test is one of the most commonly used methods for studying thermal (anti)nociception; mice or rats are placed on a hotplate until a predetermined behavioural endpoint is observed. Differences in latency time between animals reflect the differences in nociceptive thresholds (Lavich et al., 2005). Here, a 500 ml glass beaker was set on the hotplate, with temperature adjusted to 55 °C. Mice were placed one by one on the hotplate, the time for the first nocifensive response was recorded using a stopwatch then the animal was immediately removed from the hotplate. The cut off time for the test was set to 30 sec after which the animal was removed from the hotplate even if the behavioral endpoint was not reached; this was done to avoid tissue damage. The recorded responses were either licking or shaking of the hind paw, vocalization, or attempting to escape; front paw licking was not recorded as a response because it is a common grooming behavior and may have no relation to discomfort. On the day of experi­ment, mice were treated as mentioned above. After the second injection and blood glucose level recording, hotplate test was performed for each mouse within a 5-minute window of the second injection, dictated by the fast elimination kinetics of the low dose of strychnine. The investigator performing the measurements was blinded to treatment groups and to the blood glucose readings. The test latency time values were normalized to the baseline readings. One-way ANOVA was performed to detect statistically significant differences between groups. The results are reported as normalized latency time values ± standard error mean. Difference between different experi­men­tal groups was considered significant when one-way ANOVA analysis yielded p < 0.05.

*Experimental groups in Hot plate test* – The test was done on four experimental groups. All test solutions were prepared in PBS, pH 7.4. Insulin syringes (30G) were used for injection. The maximum volume of glucose injections was 0.6 ml. The maximum volume for strychnine injections was 0.3 ml. Thus, all mice received two intraperitoneal (IP) injections within 30 min. Control group injection was two times PBS (vehicle); glucose group was 2 g/kg glucose before PBS; strychnine group was PBS before 0.2 mg/kg strychnine and finally glucose + strychnine group was 2 g/kg glucose before 0.2 mg/kg strychnine.

**References**

Ayala, J. E., Samuel, V. T., Morton, G. J., Obici, S., Croniger, C. M., Shulman, G. I., Wasserman, D. H., & McGuinness, O. P. (2010). Standard operating procedures for describing and performing metabolic tests of glucose homeostasis in mice. *Dis Model Mech., 3*(9-10), 525-534. doi: 10.1242/dmm.006239

Bradman, M. J., Ferrini, F., Salio, C. , & Merighi, A. (2015). Practical mechanical threshold estimation in rodents using von frey hairs/semmes-weinstein monofilaments: Towards a rational method. *J Neurosci Methods. 255*, 92-103. doi: 10.1016/j.jneumeth.2015.08.010

Cer, R. Z., Mudunuri, U., Stephens, R., & Lebeda, F. J. (2009) IC50-to-Ki: a web-based tool for converting IC50 to Ki values for inhibitors of enzyme activity and ligand binding. *Nucleic Acids Res. 37,* W441-W445. doi: 10.1093/nar/gkp253

Chaplan, S. R., Bach, F. W. , Pogrel, J. W., Chung, J. M. , & Yaksh, T. L. (1994). Quantitative assessment of tactile allodynia in the rat paw. *J. Neurosci Methods., 53*(1), 55-63.

Dixon, W. J. (1965). The up-and-down method with small samples. *J Am Stat Assoc., 60*(312), 967. doi: 10.2307/2283398.

Lavich, T. R., Cordeiro, R. S., Silva, P. M., & Martins, M. A. (2005) A novel hot-plate test sensitive to hyperalgesic stimuli and non-opioid analgesics. *Braz J Med Biol Res 38*(3), 445-451. doi: /S0100-879X2005000300016.

Maiaru, M., Tochiki, K. K., Cox, M. B., Annan, L. V., Bell, C. G., Feng, X., Hausch, F., & Geranton, S. M. (2016) The stress regulator FKBP51 drives chronic pain by modulating spinal glucocorticoid signaling. *Sci Transl Med. 8*(325), p. 325ra19. doi: 10.1126/ scitranslmed.aab3376.

**Supplementary Material S2**

**Blood Glucose Levels and Paw Withdrawal Thresholds of Mice**

Blood glucose concentrations for each experimental group are listed. Blood glucose was determined using a commercial glucometer before and 30 min after ip injection of 2 g/kg of glucose. Control groups received an injection of vehicle (sterile PBS) only.

The ratio (PWT before/after injections of glucose and strychnine) of paw withdrawal thresholds for each animal are also given.

Means ± SEM data were used to construct Figures 3B and 3D.

**Supplementary Table S1**

|  | **Control – vehicle only** | | | | **Glucose 2 g/kg – no strychnine** | | | |
| --- | --- | --- | --- | --- | --- | --- | --- | --- |
|  | **BGL  before mg/dl** | **BGL   30 min mg/dl** | **BGL  fold change** | **PWT ratio** | **BGL  before mg/dl** | **BGL  30 min mg/dl** | **BGL  fold change** | **PWT ratio** |
| 1 | 170 | 111 | 0.65 | 1.00 | 139 | 321 | 2.31 | 1.64 |
| 2 | 151 | 112 | 0.74 | 1.55 | 225 | 580 | 2.58 | 2.25 |
| 3 | 149 | 153 | 1.03 | 1.10 | 165 | 371 | 2.25 | 4.92 |
| 4 | 131 | 128 | 0.98 | 1.12 | 147 | 287 | 1.95 | 2.80 |
| 5 | 107 | 86 | 0.80 | 0.78 | 58 | 84 | 1.45 | 1.34 |
| 6 | 120 | 139 | 1.16 | 1.30 | 157 | 136 | 0.87 | 2.70 |
| 7 | 133 | 172 | 1.29 | 0.75 | 138 | 224 | 1.62 | 1.07 |
| 8 | 147 | 178 | 1.21 | 2.09 | 107 | 174 | 1.63 | 1.25 |
| 9 | 138 | 116 | 0.84 | 0.81 | 122 | 120 | 0.98 | 3.46 |
| 10 | 120 | 112 | 0.93 | 1.06 | 114 | 216 | 1.89 | 0.37 |
| 11 | 151 | 134 | 0.89 | 0.87 | 119 | 434 | 3.65 | 0.93 |
| 12 | 183 | 127 | 0.69 | 1.63 | 95 | 237 | 2.49 | 0.71 |
| 13 | 104 | 157 | 1.51 | 0.84 | 165 | 309 | 1.87 | 1.92 |
| 14 | 144 | 102 | 0.71 | 2.78 | 110 | 285 | 2.59 | 2.14 |
| 15 | 171 | 138 | 0.81 | 2.29 | 129 | 317 | 2.46 | 3.23 |
| 16 | 86 | 94 | 1.09 | 0.48 | 113 | 247 | 2.19 | 1.59 |
| 17 | 112 | 152 | 1.36 | 0.90 | 124 | 264 | 2.13 | 2.20 |
| 18 | 108 | 181 | 1.68 | 0.87 | 132 | 163 | 1.23 | 0.86 |
| 19 | 123 | 129 | 1.05 | 0.55 | 105 | 174 | 1.66 | 1.87 |
| 20 | 101 | 102 | 1.01 | 0.33 | 111 | 201 | 1.81 | 3.08 |
| 21 | 127 | 150 | 1.18 | 0.78 | 135 | 231 | 1.71 | 0.64 |
| 22 |  |  |  |  | 91 | 189 | 2.08 | 5.76 |
| 23 |  |  |  |  | 103 | 130 | 1.26 | 5.54 |
| 24 |  |  |  |  | 103 | 160 | 1.55 | 1.26 |
| 25 |  |  |  |  | 125 | 256 | 2.05 | 2.85 |
| 26 |  |  |  |  | 101 | 191 | 1.89 | 1.12 |
| 27 |  |  |  |  | 139 | 265 | 1.91 | 2.15 |
| 28 |  |  |  |  | 70 | 228 | 3.26 | 5.76 |
| 29 |  |  |  |  | 123 | 243 | 1.98 | 5.54 |
| 30 |  |  |  |  | 70 | 228 | 3.26 | 1.20 |
|  |  |  |  |  |  |  |  |  |
| **avg** | **132** | **132** | **1.03** | **1.14** | **121** | **242** | **2.02** | **2.40** |
| **SD** | **25** | **27** | **0.27** | **0.62** | **32** | **99** | **0.64** | **1.62** |
| **SEM** | **6** | **6** | **0.06** | **0.13** | **6** | **18** | **0.12** | **0.30** |

**Supplementary Table S1|** Blood glucose levels and Pain withdrawal thresholds (PWT) – control group

**Supplementary Table S2**

|  | **no glucose – Strychnine 0.2 mg/kg** | | | | **Glucose 2 g/kg – Strychnine 0.2 mg/kg** | | | |
| --- | --- | --- | --- | --- | --- | --- | --- | --- |
|  | **BGL  before mg/dl** | **BGL   30 min mg/dl** | **BGL  fold change** | **PWT ratio** | **BGL  before mg/dl** | **BGL  30 min mg/dl** | **BGL  fold change** | **PWT ratio** |
| 1 | 165 | 371 | 2.25 | 0.48 | 139 | 321 | 2.31 | 0.69 |
| 2 | 172 | 158 | 0.92 | 0.62 | 225 | 580 | 2.58 | 0.64 |
| 3 | 121 | 126 | 1.04 | 0.87 | 110 | 167 | 1.52 | 2.40 |
| 4 | 135 | 170 | 1.26 | 0.24 | 161 | 210 | 1.30 | 4.47 |
| 5 | 116 | 101 | 0.87 | 0.76 | 81 | 76 | 0.94 | 1.66 |
| 6 | 141 | 75 | 0.53 | 0.15 | 190 | 172 | 0.91 | 1.52 |
| 7 | 132 | 190 | 1.44 | 0.98 | 114 | 138 | 1.21 | 0.59 |
| 8 | 118 | 145 | 1.23 | 0.73 | 122 | 272 | 2.23 | 0.48 |
| 9 |  |  |  |  | 120 | 134 | 1.12 | 4.34 |
| 10 |  |  |  |  | 102 | 356 | 3.49 | 1.02 |
| 11 |  |  |  |  | 103 | 281 | 2.73 | 1.46 |
| 12 |  |  |  |  | 81 | 175 | 2.16 | 0.86 |
| 13 |  |  |  |  | 128 | 134 | 1.05 | 0.93 |
| 14 |  |  |  |  | 164 | 288 | 1.76 | 1.28 |
|  |  |  |  |  |  |  |  |  |
| **avg** | **138** | **167** | **1.19** | **0.60** | **131** | **236** | **1.81** | **1.59** |
| **SD** | **21** | **90** | **0.51** | **0.30** | **41** | **129** | **0.79** | **1.30** |
| **SEM** | **7** | **32** | **0.18** | **0.10** | **11** | **34** | **0.21** | **0.35** |

**Supplementary Table S2|** Blood glucose levels and Pain withdrawal thresholds (PWT) – 0.2 mg/kg Strychnine group

**Supplementary Table S3**

|  | **no glucose – Strychnine 0.5 mg/kg** | | | | **Glucose 2 g/kg – Strychnine 0.5 mg/kg** | | | |
| --- | --- | --- | --- | --- | --- | --- | --- | --- |
|  | **BGL  before mg/dl** | **BGL   30 min mg/dl** | **BGL  fold change** | **PWT ratio** | **BGL  before mg/dl** | **BGL  30 min mg/dl** | **BGL  fold change** | **PWT ratio** |
| 1 | 120 | 176 | 1.47 | 0.17 | 135 | 320 | 2.37 | 0.82 |
| 2 | 93 | 126 | 1.35 | 1.46 | 104 | 230 | 2.21 | 4.05 |
| 3 | 78 | 90 | 1.15 | 0.19 | 100 | 97 | 0.97 | 0.67 |
| 4 | 83 | 91 | 1.10 | 0.30 | 98 | 203 | 2.07 | 0.38 |
| 5 | 86 | 78 | 0.91 | 0.14 | 98 | 204 | 2.08 | 1.50 |
| 6 |  |  |  |  | 62 | 87 | 1.40 | 0.15 |
| 7 |  |  |  |  | 85 | 228 | 2.68 | 0.31 |
| 8 |  |  |  |  | 64 | 131 | 2.05 | 0.50 |
|  |  |  |  |  |  |  |  |  |
| **avg** | **92** | **112** | **1.20** | **0.45** | **93** | **188** | **1.98** | **1.05** |
| **SD** | **17** | **40** | **0.22** | **0.57** | **23** | **78** | **0.54** | **1.28** |
| **SEM** | **7** | **18** | **0.10** | **0.25** | **8** | **28** | **0.19** | **0.45** |

**Supplementary Table S3|** Blood glucose levels and Pain withdrawal thresholds (PWT) – 0.5 mg/kg Strychnine group
